# Cognitive Intervention Through Photo-Integrated Conversation Moderated by Robots (PICMOR) Program: A Randomized Controlled Trial

**DOI:** 10.3389/frobt.2021.633076

**Published:** 2021-04-12

**Authors:** Mihoko Otake-Matsuura, Seiki Tokunaga, Kumi Watanabe, Masato S. Abe, Takuya Sekiguchi, Hikaru Sugimoto, Taishiro Kishimoto, Takashi Kudo

**Affiliations:** ^1^ Center for Advanced Intelligence Project, RIKEN, Chuo-ku, Japan; ^2^ Department of Neuropsychiatry, School of Medicine, Keio University, Tokyo, Japan; ^3^ Department of Psychiatry, Graduate School of Medicine, Osaka University, Suita, Japan

**Keywords:** cognitive intervention, cognitive decline, social interaction, social activity, robot

## Abstract

Social interaction might prevent or delay dementia, but little is known about the specific effects of various social activity interventions on cognition. This study conducted a single-site randomized controlled trial (RCT) of Photo-Integrated Conversation Moderated by Robots (PICMOR), a group conversation intervention program for resilience against cognitive decline and dementia. In the RCT, PICMOR was compared to an unstructured group conversation condition. Sixty-five community-living older adults participated in this study. The intervention was provided once a week for 12 weeks. Primary outcome measures were the cognitive functions; process outcome measures included the linguistic characteristics of speech to estimate interaction quality. Baseline and post-intervention data were collected. PICMOR contains two key features: 1) photos taken by the participants are displayed and discussed sequentially; and 2) a robotic moderator manages turn-taking to make sure that participants are allocated the same amount of time. Among the primary outcome measures, one of the subcategories of cognitive functions, verbal fluency significantly improved in the intervention group. Among the process outcome measures, a part of the subcategories of linguistic characteristics of speech, the amount of speech and richness of words, proportion of providing topics, questions, and answers in total utterances were larger for the intervention group. This study demonstrated for the first time the positive effects of a robotic social activity intervention on cognitive function in healthy older adults via RCT. The group conversation generated by PICMOR may improve participants’ verbal fluency since participants have more opportunity to provide their own topics, asking and answering questions which results in exploring larger vocabularies. PICMOR is available and accessible to community-living older adults.

**Clinical Trial Registration:** UMIN Clinical Trials Registry, identifier UMIN000036667.

## Introduction

Cognitive health is a key component of healthy aging. Interventions for risk factors may delay or prevent a third of dementia cases (Livingston et al., 2017). While a systematic review found that social activity intervention may help maintain cognitive function among healthy older adults (Kelly et al., 2017), there are no global recommendations for social activity interventions related to cognitive health because evidence of social activity intervention’s impact is limited (World Health Organization, 2019). Thus, determining the effectiveness of social activity intervention on cognitive health is necessary.

Among the social activity interventions that exist, group-based conversation is the type that is expected to affect cognitive function in particular. Group conversation is a fundamental part of social interaction. Cohort analysis suggests that weekly verbal interactions are associated with verbal learning and memory (Zuelsdorff et al., 2019). Group sessions of cognitive stimulation therapy have also been shown to improve cognition in patients with mild-to-moderate dementia (Woods et al., 2012). However, group conversation interventions’ effects on the cognitive functions of healthy older adults are unclear. This is in part because of the difficulty in validating the effects of group conversation, a difficulty resulting from the fact that participation in a group conversation is not easy for older adults who have sensory deficits and/or decrements in language comprehension and production (Gerontological Society of America, 2012). Cognitive changes in older adults are highly variable from person to person, which may also lead to diversity in the level and manner of participation in a group conversation and of outcomes. If there is enough of an imbalance in the amount of speech for the participants, the participants may end up participating in functionally different cognitive tasks. However, no study so far has measured the manner of participation in a group conversation, what conditions regulate it, or its differential effects on cognition in healthy older adults.

Compared to exercise interventions, conversation intervention studies are still at the embryonic phase. For exercise intervention, aerobic training, resistance training, and multimodal training have been tested, and their effects have been compared (Barha et al., 2017). While exercise intervention can set intensity, such as the weighting levels for resistance training, conversation interventions have not done this since conversations are more spontaneous, and regulating intensity beforehand is problematic. The characteristics of conversation that may prove effective have not been clarified nor even quantified yet. Merely, results have been summarized stating that the conversation lasted for a certain period of time. It has been reported that a human-type communication robot has a positive effect on cognitive function in elderly women living alone, but they report the manner of communication as “living with communication robots for 8 weeks” (Tanaka et al., 2012). The manner and even quantity of conversation with the robot has not been reported. In the pioneering study on web-enabled conversation intervention, some of the conversation sessions were transcribed (Dodge et al., 2015). There, the number of spoken words contributed by the participant or interviewer serves as a metric to improve the standardization of the individual interviewers' interview skills. However, the number of words spoken by each participant during conversation sessions has not been reported.

Research on technology and aging is growing (Pruchno, 2019), with some studies considering tools and interventions for social connectivity and reduced loneliness (Czaja et al., 2018), well-being (Pu et al., 2019), and physical, social, and cognitive activity (Croff et al., 2019). Narrative review indicated that social robot interactions could improve engagement, interaction, and stress indicators, as well as reduce loneliness and the use of medications for older adults (Pu et al., 2019). The results of the meta-analysis suggest that pet robot intervention may be suitable as a treatment option for BPSD in people with dementia (Leng et al., 2018). Inspired by these initiatives, we propose Photo-Integrated Conversation Moderated by Robots, which contains two key technologies: 1) a group conversation support method called Coimagination (Otake et al., 2011; Otake-Matsuura, 2018), in which each participant is allocated an equal amount of time for talking, listening, and question and answer time, and prepares topics and takes photos beforehand according to sessional themes; and 2) a robot that measures each participant’s speech and supports turn-taking on that basis during the discussion phase of the intervention (Yamaguchi et al., 2012). The Coimagination method follows the recommendations (Gerontological Society of America, 2012), which are oriented to eliminate older adults’ communication difficulties with healthcare professionals, but they are applicable to the communication among older adults as well. Specific recommendations are the use of photos as supports, giving each participant equal opportunity to talk, and verifying comprehension through question and answer sessions.

The design of the robot-guided intervention proposed here—specifically, the control on the amount of speech from each participant—is based on the recommendation that each participant has equal opportunity to talk. We assume that the amount of speech or the number of words in each conversation intervention is a fundamental variable representing intensity, which is equivalent to the amount of weight in resistance training. This paper addresses the lack of quantification of conversation intervention in the existing studies. The objectives here are to propose PICMOR as a protocol designed to guarantee the intensity of conversation intervention and then to discuss the effect on cognition in the light of linguistic characteristics.

This study’s purpose was to gather evidence of the effects of PICMOR on cognition in healthy older adults, and to validate PICMOR using a randomized controlled trial (RCT). We will discuss the effects and their possible sources. Group conversation without guidance or feedback was used in the control group; conversation was encouraged in both groups (instead of using a control group with less conversation) to allow variation in speech amounts among participants to emerge and examine the possibility that balanced speech may have positive effects on the cognition of older adults, in particular, verbal production and comprehension.

This paper first focuses on the primary and secondary outcome measures of the trial, that is, cognitive functions and quality of life. Then, we explore process outcome measures: linguistic characteristics of speeches in both groups to compare interaction quality, and the number of photos taken and memory recall scores of the intervention to estimate engagement. Our hypothesis is that participants who complete the PICMOR intervention will show subsequent improvement on certain subcategories of cognitive functions and quality of life from baseline to post-treatment compared to participants in the active control program.

## Materials and Methods

### Study Design and Procedures

**FIGURE 1:**
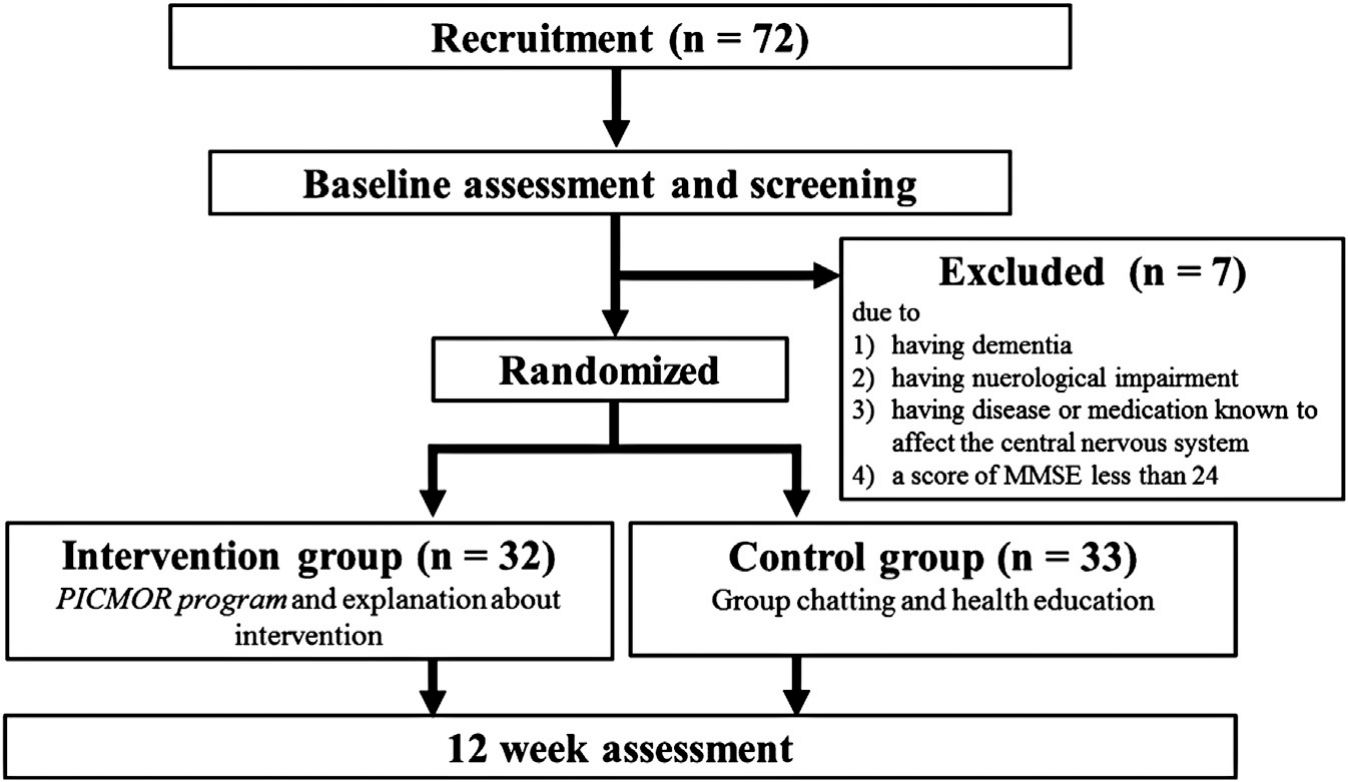
CONSORT diagram flowchart.

The 12-week RCT took place from June to September 2018 (UMIN Clinical Trials Registry number: UMIN000036667). Figure 1 presents the CONSORT flowchart of this study. First, screening and baseline assessment (medical interviews, neuropsychological tests, and self-reported questionnaires) were conducted to determine participant eligibility. After baseline assessment, participants were randomly assigned to the intervention or control group according to the Japanese version of the Mini-Mental State Examination (MMSE-J) scores (Sugishita et al., 2018) and Japanese version of Montreal Cognitive Assessment (MoCA-J) scores (Fujiwara et al., 2010). After 12 weeks of intervention, a post-assessment was conducted. There were no drop-outs to follow-up during the intervention. The assessors were not involved in the intervention delivery.

Intervention group participants received weekly 30 min intervention sessions, each followed by 30 min of explanation about the intervention. The active control sessions in the control group involved 30 min of weekly unstructured conversation among the group and 30 min of health education about successful aging. The common instruction to both groups is “Please talk as usual as possible although you do not know each other in the beginning.” Each group was divided into four-person subgroups with both men and women, formed on the basis of participants’ availability.

The Institutional Review Board approved this study. All participants provided written informed consent. This study was registered after the onset of participant enrollment.

### Participants

The participants were community-living healthy adults aged over 65 years, recruited from the Silver Human Resources Center. The exclusion criteria were as follows: dementia; neurological impairment; any disease or medication known to affect the central nervous system; MMSE-J score less than 24. Seventy-two people received screening.

### Intervention: Photo-Integrated Conversation Moderated by Robots

**FIGURE 2:**
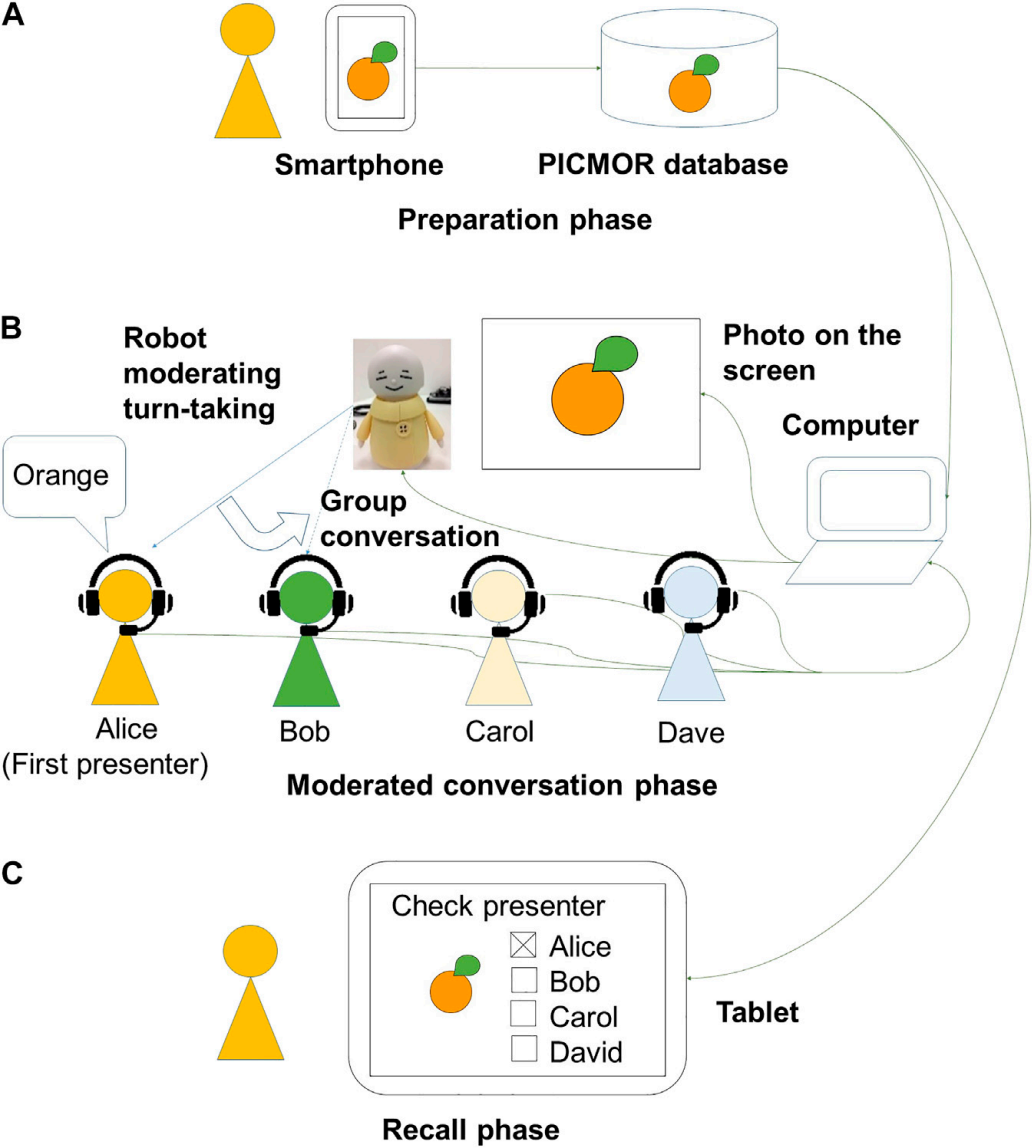
Experimental setup for Photo-Integrated Conversation Moderated by Robots (PICMOR).

**FIGURE 3:**
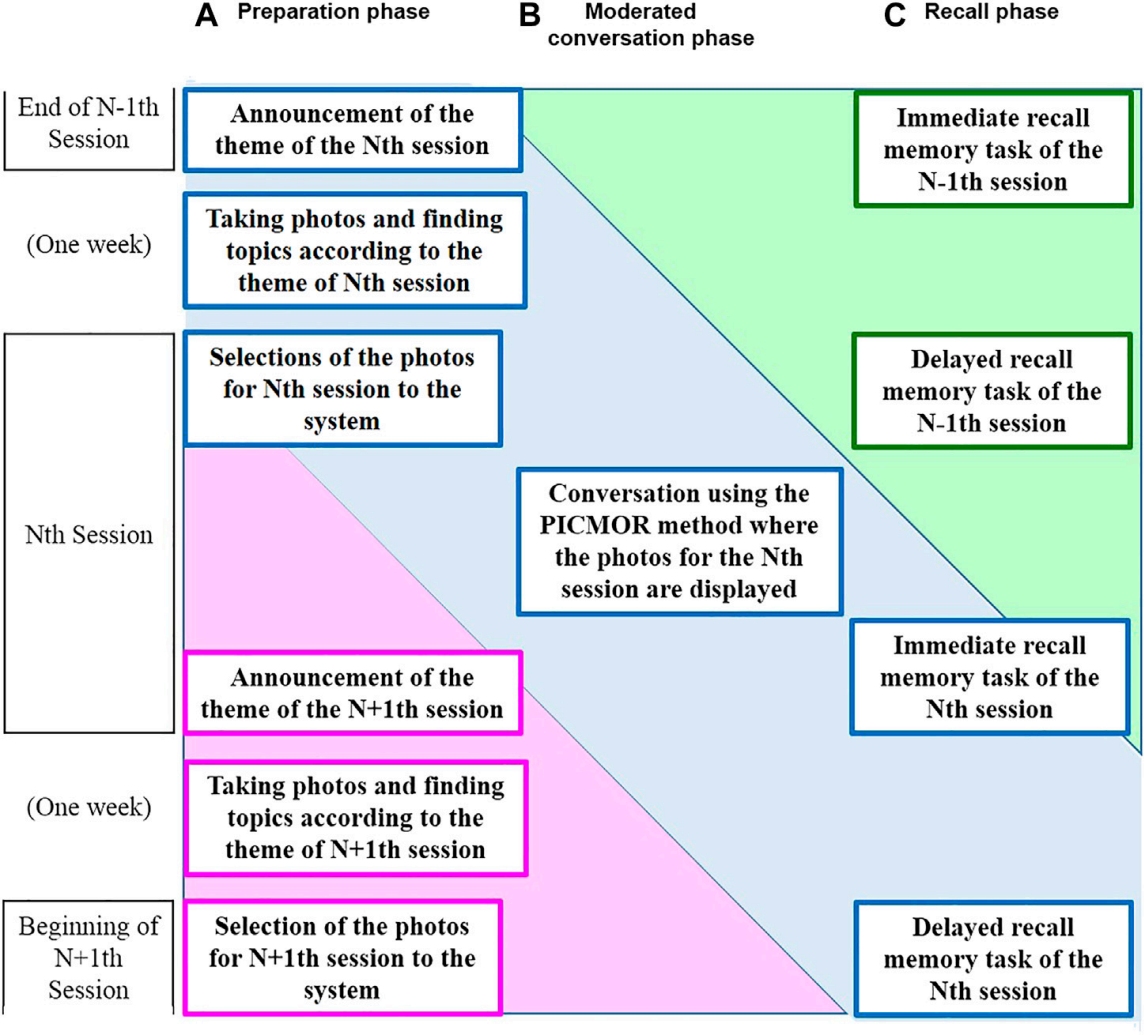
A block diagram of the RCT based on the PICMOR program.

PICMOR is an integrative intervention program supporting the preparation of conversation topics, time management, and turn-taking in conversations, and reflection on the topics. The PICMOR program consists of three phases: preparation, conversation, and recall (see Figure 2). A block diagram of RCT based on the PICMOR program is shown in Figure 3. The main phase is conversation. In order to make participants well-prepared and focused during the conversation, preparation and recall phases precede and follow the conversation, respectively.

#### Preparation Phase

During this first phase, each participant used a smartphone with a specially developed application to take photos that represent topics related to a theme. We tested the application beforehand to ensure that older adults using smartphones for the first time would be comfortable. The initial screen of the application comprised only two buttons: one to take a photo, and another to select photos for conversation. Photos were smoothly uploaded to the online PICMOR database (see Figure 2A). The participants’ assignment was to take photos and find topics based on a set theme of the week for participants to talk about. When the theme of the session was “Favorite Food,” for example, the topic of the first presenter, Alice, was “Orange.” The theme is announced before a given week’s session. Since the program was provided weekly, the theme for the following week’s session was announced to participants at the end of each session. As shown in Figure 3, the theme of the Nth session is announced at the end of the N − 1th session, and the theme of the N + 1th session is announced at the end of the Nth session. In this study, each participant engaged with 12 themes because he/she has participated for 12 weeks in the experiment. The 12 themes are shown in Table 1. The themes were designed to trigger activities that produce new episodic memories, and enhance attention and/or planning functions. For instance, the theme, “Found on a 10 min walk” was selected to trigger activities that produce new episodic memories. The theme, “Seasonal things,” was selected to enhance attention to environments. The theme, “Cleaning, before and after” was selected to enhance planning functions. The location, timing, and frequency of taking photos depended on the participant. They were asked to take as many photos as possible and select the best two in the beginning of the session (in the middle on the left of Figure 3). In total, 24 photos were used for each participant during the 12 weeks of intervention. The photos were used rather than video clips or sounds because participants can talk while the photos are displayed during the moderated conversation phase.

**TABLE 1:** The themes for each session.

Session	Theme
1	Favorite things
2	Neighborhood landmarks
3	Feeling the season
4	Favorite foods
5	For my health
6	Tips for daily living
7	Being mindful of disaster prevention
8	Funny stories and mistakes
9	Things to get rid of
10	Found on a 10 min walk
11	Cleaning, before and after
12	Starting something new

#### Moderated Conversation Phase

**FIGURE 4:**
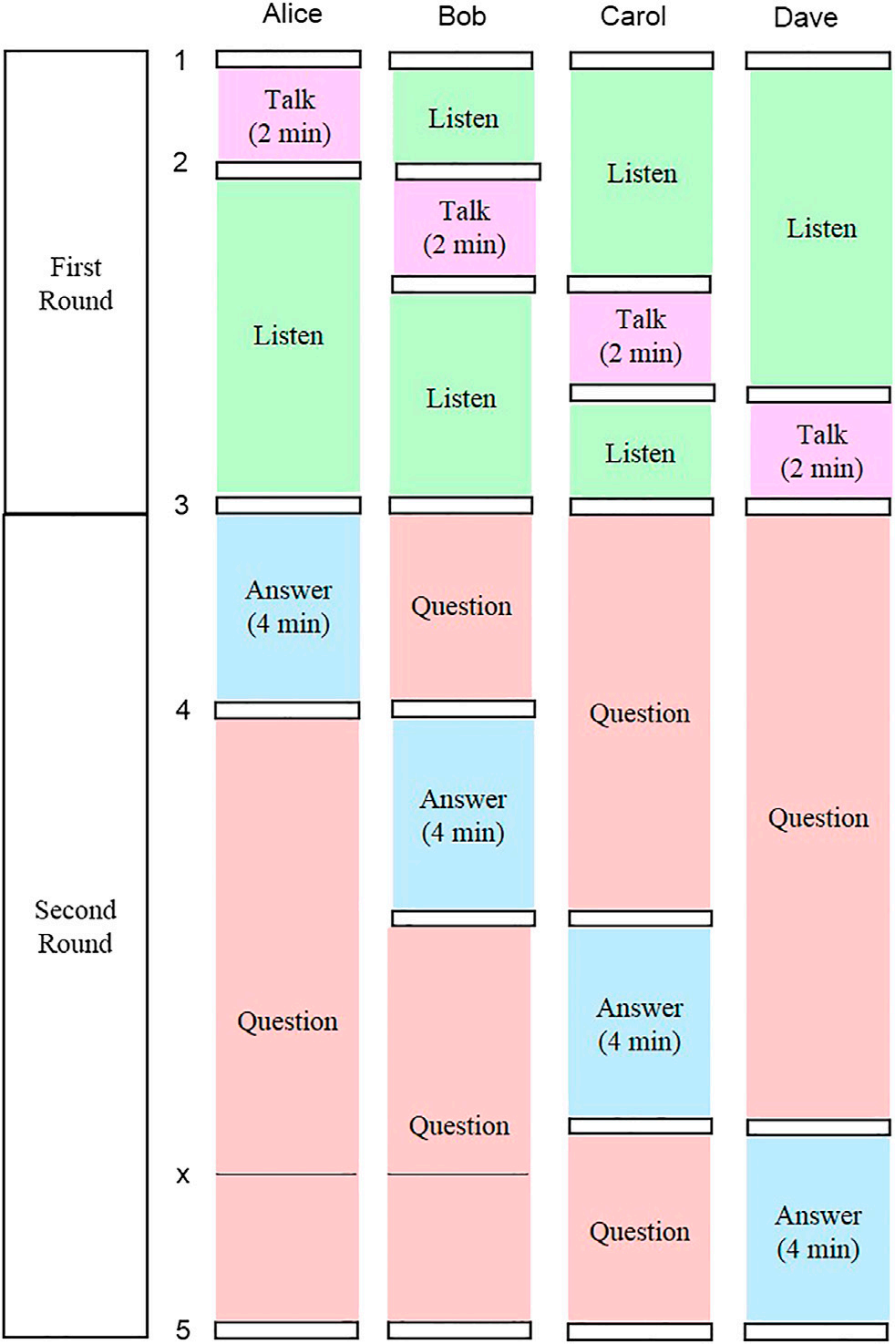
A block diagram of the moderated conversation phase in the PICMOR program.

In the second phase, participants are cued to talk when their photos are displayed on the screen. Each set of photos is displayed sequentially, and each set is presented twice. In the first round, all participants describe their own photos; in the second round, they discuss each other’s photos (see Figure 2B, where participants are in a conversation setting using the Coimagination method, looking at their photos). A block diagram of the moderated conversation phase is shown in Figure 4. Participants were divided into groups of four people. The conversation session consisted of two stages. First, each participant was assigned 2 min to present two photos related to the topic, each of which was displayed for 1 min (see, upper part of Figure 4). Second, during the discussion, the other participants asked questions and gave comments to the presenter. Each participant was assigned 4 min for discussion, during which each photo was displayed for 2 min (see, lower part of Figure 4). This process was repeated for each participant. Each session lasted about 30 min, including the instructions given by a robot and the interval between each presentation and discussion. The instructions are shown in Table 2. The numbers and the letter in the table refer to the timing illustrated in Figure 4. The first instruction, “Now, it’s time to start a conversation session.” is given in the beginning of the first round. The fifth instruction, “Thank you very much for active conversation to you all.” is given at the end of the second round. The instruction named “x” is given occasionally when the imbalance of the amount of speech among participants is observed.

**TABLE 2:** Instructions given by the robot.

Timing	Instructions
1	Now, it’s time to start a conversation session.
	Today’s theme is “Favorite food.”
	Each participant describes 2 photos.
	Please give a talk in 2 min and ask questions in 4 min for each.
	Now, it’s time to give a talk.
	Alice, please talk about your photos.
2	Thank you very much.
	Bob, please talk about your photos.
3	Thank you very much.
	Now, it’s time to ask questions and give comments.
	Please ask questions and give comments to Alice.
4	Thank you very much.
	Please ask questions and give comments to Bob.
5	Thank you very much for active conversation to you all.
	It’s time to close the conversation session.
X	Alice, thank you very much.
	Bob, how about you?

Conversations were moderated by a “chair-robot” developed by us. Named Bono-05, this robot is proficient at time management (Otake-Matuura and Tokunaga, 2020). A robot was chosen as moderator instead of a human, as a robot has the skills to apply turn-taking moderation based on speech pattern analysis, which a human does not. Bono-05 has four degrees-of-freedom—head pitching, body rotation, and left and right arm elevations—which are sufficient for its moderator role. For instance, when a speaker finishes talking, the robot is able to select the next speaker by rotating its body at a given angle and lifting its right arm horizontally to address that person directly. With a loud speaker installed inside of the main body of the robot, Bono-05 can say to a participant, “Please talk about your photos.,” which is included in the first and second instruction in Table 2. In this study, Bono-05 automatically and strictly moderated the conversation and conducted turn-taking based on the time slot duration previously determined by the researchers. A robot possesses a range of skills that a human moderator would not: during the discussion, the robot would prompt and stop participants’ utterances automatically based on total speech time, silent time, and utterance length so as to balance the production of speech for each participant (Yamaguchi et al., 2012); when the robot detected that a participant had spent less time than the others on conversation, it would directly encourage the participant to provide questions or comments. For instance, when Alice speaks too much and Bob speaks the least, the robot may say “Alice, thank you very much. Bob, how about you?,” which is on the bottom of Table 2. Each participant wore a headset-microphone that recorded his/her voice to measure each participant’s speech precisely. This audio data was transmitted in real-time from the microphones to a computer via cables to precisely measure and balance the amounts of speech and to transcribe the speech for linguistic analysis. In this study, we also recorded videos to capture the details of each conversation. Participants were filmed from behind to protect their privacy.

#### Recall Phase

In the last phase, the participants completed memory tasks using a specially developed tablet application. The photos previously displayed during the conversation were randomly shown, and the participants were asked to indicate the presenter who took the photo by touching the name on the touch panel (see Figure 2C. These tasks were conducted at two points in time: soon after the end of the conversation in order to measure immediate recall and one week later in order to measure delayed recall. The immediate recall task was meant to check if the participants could focus on listening and understanding, resulting in remembering the presenter’s name of each photo. The delayed recall task was conducted just before the next conversation of the week to check whether the participant’s memories were preserved. The immediate recall task of the N − 1th session is done at the end of the N − 1th session, and the delayed recall task of the N − 1th session is done in the beginning of the Nth session. The immediate recall task of the Nth session is done at the end of the Nth session, and the delayed recall task of the Nth session is done in the beginning of the N + 1th session, shown on the right of Figure 3.

### Outcome Measures

The primary outcome measures in the present study were cognitive performance measures evaluated by standardized neuropsychological tests conducted by well-trained examiners. The following tests were selected hypothesizing that the conversational intervention would lead to improving memory, attention, executive function since conversations are reported to require these functions (Ybarra et al., 2008; Ybarra and Winkielman, 2012).

The MMSE-J (Sugishita et al., 2018.) and MoCA-J (Fujiwara et al., 2010) were administered to evaluate global cognitive function. The logical memory subtests, Logical Memory I and II, from the Wechsler Memory Scale-Revised (WMS-R) (Wechsler, 1987) were introduced to evaluate memory function. Logical Memory I assesses immediate recall of the content of a story immediately after the examiner reads it, while Logical memory II assesses delayed recall 30 min later. Two kinds of stories were used, one chosen randomly at base assessment and another used at post-assessment. The Advanced Trail Making Test (ATMT) (Mizuno and Watanabe, 2008) assesses attention and executive functions using a computer. The Wechsler Adult Intelligence Scale—Third Edition (WAIS-iii) (Wechsler, 1997), Digit Span Forward and Backward, and Digit Symbol Coding tests were also used. Digit Span Forward assesses simple memory span, and Digit Span Backward assesses working memory capacity. The Digit Symbol Coding test assesses the process speed and memory in digit symbol coding performance, which requires the subject to write down each corresponding symbol as fast as possible. In the verbal fluency tests, letter fluency was evaluated to measure verbal function; specifically, participants were asked to pronounce as many words as possible starting with the Japanese character “ka” in 1 min, and then the total number of words was counted.

The secondary outcome measures covered subjective physical and mental status and quality of life. The Tokyo Metropolitan Institute of Gerontology-Index of Competence (TMIG-IC) (Koyano et al., 1991), the Japanese version of the Geriatric Depression Scale short form (GDS-15-J) (Sugishita et al., 2017), and the WHO Quality of Life questionnaire 26 (WHO QOL 26) (Tazaki and Nakane, 1997) were used.

The process outcome measures were the linguistic characteristics of speeches in both groups to compare the quality of interactions, and the number of photos taken and memory recall scores to estimate engagement in the intervention group. Linguistic characteristics were selected as the process outcome measures since linguistic ability is known to be correlated with late-life changes in cognition in healthy older adults and those with dementia (Kemper et al., 2001; Riley et al., 2005; Kemper, 2009).

### Analysis

For basic characteristics at baseline, Welch’s t-test was used to compare the means of continuous variables (Age, MMSE-J, MoCA-J, GDS-15, and TMIC-IC) and Fisher’s exact test was used to compare frequency distributions of categorical variables (Gender and Education) between groups.

To estimate the intervention effects on the aforementioned outcome measures, linear mixed models with random-effect intercepts for participants were performed for all outcome measures using the “lmer” function in the R package, “lme4” (Bates et al., 2015).

Because the scores for each individual were not expected to be independent, it was not appropriate to just pool the whole sample for simple regression analyses. We indeed observed that the intraclass correlation coefficient took the maximum value of 0.86 for logarithmically transformed scores of TMTs-A, and even the minimum value—0.21 for the scores of Digit Symbol Coding—was not negligible, which suggests the non-ignorable hierarchy of the data and justifies the use of mixed models. We measured sizes of the intervention effects by the *f^2^*, which was derived by inserting the pseudo-R-squared obtained from the R package “MuMIn” (Bartoń, 2020) into the formula shown in Selya et al. (2012).

The models have the following independent variables: time (1 = post-experiment; 0 = pre-experiment), group (1 = intervention group; 0 = control group), and their interaction term time × group, which is interpreted as the intervention effect. To obtain *p*-values associated with the linear mixed analyses, we used the R package “lmerTest” (Kuznetsova et al., 2017). It was confirmed visually that there were no severe deviations from straight lines in normal quantile–quantile plots of residuals. Taking the relatively small sample size into account, we judged that the assumption of normality was reasonably valid. However, for TMIG-IC, TMTs-A and -B, large deviations from straight lines were observed. Therefore, for TMTs-A and -B, the mixed linear models were performed for logarithmic transformed outcomes. For TMIG-IC, instead of using the mixed linear model, we performed the mixed Poisson regression analysis for the number of items indicating lower functional capacity, by using the “glmer” function in the R package, “lme4” (Bates et al., 2015).

#### Analysis of Conversation

To investigate differences in conversation patterns between the control and intervention groups, we quantitatively analyzed conversation transcriptions derived from the audio data. We focused on the number of words and lexical richness (i.e., type–token ratio) after decomposing the sentences into words, rather than the meanings of conversations or words. The reason is that when we consider the cognitive function in conversation, how much information participants can express through conversation is considered important (Snowdon et al., 1996; Kemper et al., 2001). Therefore, we quantified the conversational characteristics for each participant as a simple index based on the number of unique words.

For the analysis, first, we used Google Cloud Speech-to-Text (Google, 2018) to automatically transcribe audio to text data, and then manually checked the entire text by comparing it to the audio data and fixing any mistakes. Second, we conducted morphological analysis using “MeCab” (ver. 0.996), a useful tool for Japanese morphological analysis based on conditional random fields (Kudo et al., 2004). Finally, we calculated the number of spoken words per time unit (per minute), the standard deviation of the number of spoken words in each session, and lexical richness for each participant. We used bilogarithmic type-token ratio (logTTR), defined as log(number of types)/log(number of tokens), to quantify lexical richness (Wachal and Spreen, 1973). If this value is high, the speech contains much more information in terms of the number of vocabulary items.

We also assigned tags indicating the defining characteristics of the Coimagination method to each utterance. The tags assigned were 1) topic provision, 2) question, 3) reply, and 4) others (less meaningful utterances, listening back, etc.). Then, we checked the data for any correlations between each participant’s total number of utterances and their percentages of tags to ascertain whether those who tended to provide their own topics also had a higher total number of utterances. In addition to this analysis at the individual level, we also conducted a similar analysis at the aggregate level; that is, we divided the data into two halves, top and bottom, according to the total number of utterances (hereafter referred to as the “Higher” and “Lower” subgroups, respectively). We did this with the data for both the intervention and control groups, pooled participant’s data within them, and then analyzed the differences in the proportions of the tags between the subgroups. Thus, we were able to examine the extent to which speech characteristics differ according to the total number of utterances and the conditions (i.e., intervention or control).

We conducted these analyses with R Ver. 3.4.3 (R-Core Team, 2020).

#### Analysis of Photos

We evaluated participants’ engagement in preparing topics for future conversations based on the number of photos taken per session per participant, under the assumption that more photos indicated more effort to find topics for the next conversation session. We also referred to the participants’ comments on why they took a large or small number of photos.

We evaluated the accuracy rates of the photo recall tasks. If the accuracy rate of the immediate recall task was high, we assumed that attention and short-term memory were functioning well. Similarly, if the accuracy rate of the delayed recall task was high, recent memory was estimated to be well-functioning.

## Results

Seven participants were excluded at screening because they meet our exclusion criteria. Therefore, a sample of 65 people was divided into intervention and control groups (intervention: *n* = 32, control: *n* = 33). Participants in each group were divided into eight subgroups of four to five participants. Basic characteristics of study participants at baseline are presented in Table 3. The only significant difference found between groups was a significantly higher GDS-15 score in the control group. All participants completed the program and post-measurement.

**TABLE 3:** Characteristics at baseline.

	Intervention (*N* = 32)	Control (*N* = 33)	*P*
Age (Mean ± SD)	72.97 ± 3.47	72.33 ± 2.90	0.427
Gender (Female; n, %)	16 (50.0%)	19 (57.6%)	0.622
Education (≥13 years; n, %)	20 (62.5%)	17 (51.5%)	0.455
MMSE-J (Mean ± SD)	27.97 ± 1.43	28.12 ± 1.52	0.677
MOCA-J (Mean ± SD)	25.97 ± 2.47	25.42 ± 2.66	0.395
GDS-15 (Mean ± SD)	2.09 ± 1.73	3.36 ± 2.18	0.011*
TMIG-IC			
Total score (Mean ± SD)	12.31 ± 1.03	11.88 ± 1.32	0.144
The instrumental activity of daily living (Mean ± SD)	5.00 ± 0.00	4.97 ± 0.17	0.325
Intellectual activity (Mean ± SD)	3.84 ± 0.51	3.67 ± 0.74	0.265
Social role (Mean ± SD)	3.47 ± 0.67	3.24 ± 0.83	0.231

Note: Welch’s t-tests for Age, MMSE-J, MoCA-J, GDS-15, and TMIG-IC Fisher’s exact test for Gender and Education. MMSE-J, Japanese version of the Mini-Mental State Examination, MoCA-J, Japanese version of the Montreal Cognitive Assessment; GDS-15, 15-item Geriatric Depression Scale; TMIG-IC, Tokyo Metropolitan Institute of Gerontology Index of Competence; SD, standard deviation. *p < 0.05.

### Primary and Secondary Outcomes

Table 4 summarizes cognitive test scores within participants (pre- and post-experiment) and between participants (intervention and control groups). In Logical Memory I and II and Digit Symbol tests, overall scores significantly improved after the intervention. However, there was no significant time × group interaction on the scores. Regarding MMSE-J, MoCA-J, forward and backward Digit Spans, and TMTs-A and -B, neither main effects nor interaction effects were found. In the verbal fluency test, a significant time × group interaction was obtained. The regression coefficient of time × group associated with verbal fluency was 2.024 (*f^2^* = 0.017), meaning the number of generated words between pre- and post-experiment was approximately two words more than in the control group—from 11.8 at pre-experiment to 13.6 at post-experiment—while there was little change in the control group from pre-experiment (11.4) to post-experiment (11.2). In all secondary outcomes—TMIG-IC, GDS-15, and WHO QOL26—no intervention effects were found.

**TABLE 4:** Comparison of Pre/Post cognitive test scores in intervention and control groups.

	Intervention (*n* = 32)		Control (*n* = 33)		Estimates		
	Pre Mean (SD)	Post Mean (SD)	Pre Mean (SD)	Post Mean (SD)	Time (SE, *p*)	Group (SE, *p*)	Intervention (SE, *p*)
MMSE-J	28.0	28.6	28.1	28.7	0.545	−0.152	0.111
	(1.4)	(1.8)	(1.5)	(1.2)	(0.30, 0.074)	(0.37, 0.683)	(0.43, 0.797)
MoCA-J	26.0	26.3	25.4	25.2	−0.212	0.545	0.556
	(2.5)	(2.7)	(2.7)	(2.9)	(0.44, 0.631)	(0.66, 0.413)	(0.63, 0.378)
Logical memory I (immediate)	10.2	11.3	8.3	10.8	2.515***	1.915*	−1.39
	(3.2)	(3.5)	(4.0)	(4.2)	(0.72, 0.001)	(0.93, 0.042)	(1.02, 0.178)
Logical memory II (delayed)	8.5	9.5	6.5	9.1	2.606***	2.016*	−1.606
	(3.0)	(3.5)	(3.6)	(4.3)	(0.66, 0.000)	(0.90, 0.028)	(0.93, 0.091)
Verbal fluency	11.8	13.6	11.4	11.2	−0.242	0.450	2.024*
	(3.7)	(3.5)	(4.3)	(3.7)	(0.60, 0.686)	(0.95, 0.637)	(0.85, 0.020)
Symbol^a^	55.2	66.5	52.0	61.7	9.667***	3.194	1.624
	(13.1)	(11.9)	(13.7)	(11.4)	(2.40, 0.000)	(3.14, 0.312)	(3.45, 0.640)
Digit Span forward	9.8	9.7	9.6	10.1	0.455	0.114	−0.517
	(2.0)	(1.6)	(1.7)	(1.6)	(0.26, 0.085)	(0.44, 0.796)	(0.37, 0.167)
Digit Span Backward	6.4	6.4	6.1	6.5	0.394	0.347	−0.394
	(1.7)	(1.9)	(1.5)	(1.5)	(0.28, 0.165)	(0.41, 0.399)	(0.40, 0.329)
TMT-A^b^	76.1	65.5	90.5	87.6	−0.0313^c^	−0.1053^c^	−0.0773^c^
	(36.2)	(21.1)	(58.3)	(64.5)	(0.04, 0.448)	(0.11, 0.346)	(0.06, 0.184)
TMT-B^b^	141.5	103.5	140.9	121.3	−0.0993^c^	−0.0123^c^	−0.1133^c^
	(107.5)	(42.3)	(88.5)	(61.9)	(0.07, 0.133)	(0.12, 0.918)	(0.09, 0.232)

^a^ One participant with a missing value in the intervention group was eliminated.

^b^ One participant with a missing value in the intervention group was eliminated.

^c^ Estimations for logarithmic transformed response variables.

MMSE-J, Japanese version of the Mini-Mental State Examination; MoCA-J, Japanese version of the Montreal Cognitive Assessment; TMT, Trail Making Test. *p < 0.05; ***p < 0.001.

### Process Outcomes

**FIGURE 5:**
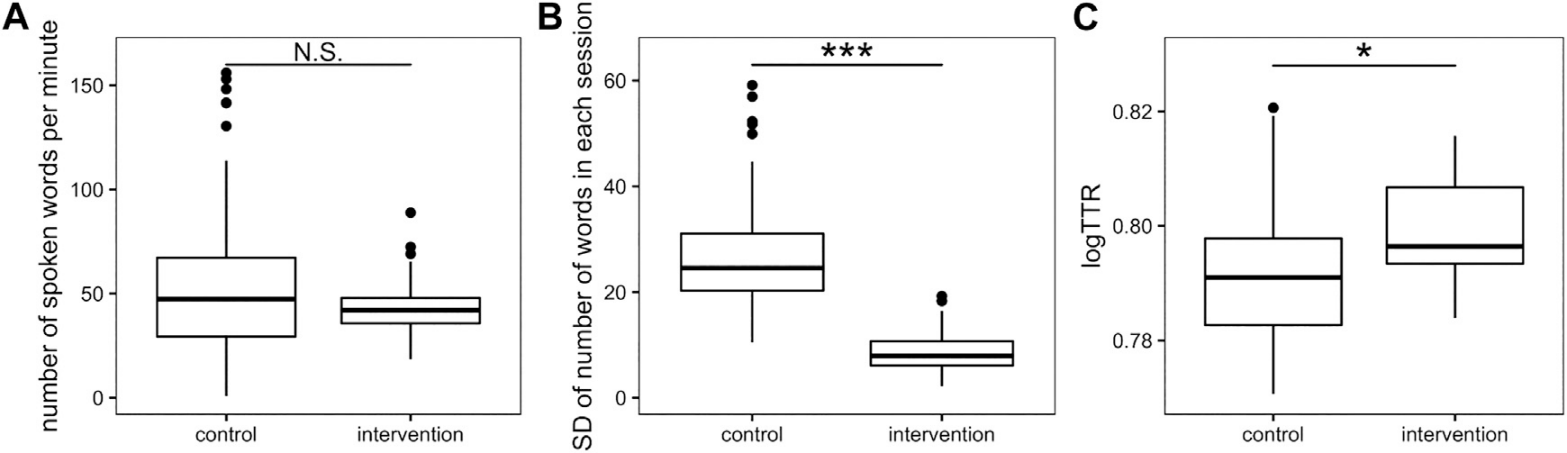
Boxplots for results of conversational analysis. **(A)** The number of spoken words per minute, **(B)** standard deviation (SD) of number of words, and **(C)** logTTR. *p < 0.05, ***p < 0.001.

We quantified conversations based on the number of words and lexical richness of the speech and thereby revealed the difference between control and intervention groups. Figure 5A shows the number of words per minute. GLMM (log link function, offset as time duration, with random effects of id and group) analysis reveals no significant difference between control and intervention in the number of words (*p* = 0.51). To investigate evenness in the number of words within groups in each session, we calculated the standard deviation of the number of words in each session (Figure 5B). The GLMM (gamma link function, random effect of group) reveals that the standard deviation in the intervention group was smaller than in the control group (*p* = 2.27 × 10^−8^), indicating that participants in the intervention group spoke more evenly than in the control group. Figure 5C shows lexical richness for each participant. The linear mixed model (random effect of group) suggests that logTTR in the intervention group was larger than in the control group (*p* = 0.029). The results demonstrate that the program intervention led the speech to contain more diverse information. Moreover, robot Bono-05 can promote and suppress speech during the session. The average number of instances of promotion and suppression of speech by the robot during each session per participant was 1.20 (SD = 1.81) and 0.95 (SD = 1.67), respectively.

**FIGURE 6:**
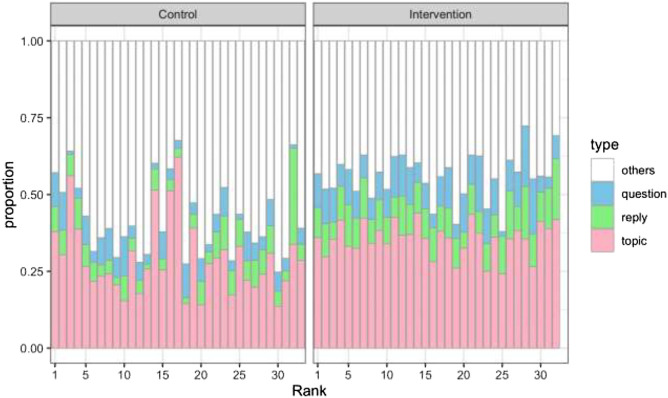
The percent stacked bar plot for four types of utterances. The horizontal axis stands for the rank order of the total number of utterances (the person on the left has the most utterances and the person on the right has the least) within the control and intervention groups.

Supplementary Table S1 presents the total number of utterances per participant and the breakdown of the tags. The percentage-stacked bar plot for the data is shown in Figure 6. In the analysis at the individual level for this data, we found no clear tendency for those who tended not to provide topics to also have a lower total number of utterances. However, suggestive results were revealed at the aggregate level. As shown in Table 5, which gives the proportions of the four types of tags within the intervention and control groups, the intervention group has higher percentages of topic provision, question tags, and reply tags. This tendency holds, moreover, even among the subgroups of participants with the fewer utterances in the intervention and control groups. In the intervention group, the differences in the percentages of these tags between the Higher and Lower subgroups is small. Notably, the percentage of questions is even higher in the Lower subgroup. We implemented Fisher's exact tests to compare the differences in proportions of the three focal tags (i.e., topic provision, question, and reply) and their complements within the subgroups of intervention and control groups, namely, 1) the Higher and Lower subgroups of the control group, 2) the Higher and Lower subgroups of the intervention group, 3) the Higher subgroups of the control and intervention groups, and 4) the Lower subgroups of the control and intervention groups. All the differences were significant at the 5% level, except for the reply tag in the case of 2).

**TABLE 5:** The numbers and proportions of four types of utterances within the subgroups.

	Control		Intervention	
	Higher	Lower	Higher	Lower
	*n* = 17	*n* = 16	*n* = 16	*n* = 16
Topic	11582	4382	7475	5629
	33.2%	25.1%	36.3%	34.6%
Question	2336	1014	1952	1712
	6.7%	5.8%	9.5%	10.5%
Reply	2010	1307	2056	1697
	5.8%	7.5%	10.0%	10.4%
Others	18930	10774	9121	7209
	54.3%	61.6%	44.3%	44.4%
Total	34858	17477	20604	16247
	100.0%	100.0%	100.0%	100.0%

All participants in the intervention group successfully used smartphones to take photos, although for 62.5% of them, it was their first time using a smartphone. The number of photos per session per participant ranged from 2 to 116 (M = 15.43, SD = 14.74). Participants who took many photos mentioned that they were interested in or excited about taking photos, while those who took few photos said that they were busy. The total number of photos taken was 5,924. The average scores on immediate and delayed recall tasks were 98.88 and 97.60%, respectively.

## Discussion

While effective interventions that improve cognitive health in aging populations are increasingly necessary (World Health Organization, 2019), evidence regarding the impact of social activity interventions on cognitive functioning remains limited. This study advances contemporary knowledge regarding the impact of social activity intervention in helping older adults participate in group conversation equally. Through a RCT, this study is one of the first to demonstrate the positive effects of social activity intervention—a group conversation regulated by a robot—on cognitive function in healthy older adults. A systematic review on robotic technologies in assisting older adults (Shishehgar et al., 2019) include only one literature which reports that the communication robot is effective for improving cognitive functions, namely, MMSE, judgement, and verbal memory (Tanaka et al., 2012). Robots assisting older adults with cognitive impairment or dementia have been studied but they didn’t aim at improving cognitive functions.

This RCT examined cognitive functions as primary outcomes for both the control and experimental groups, linguistic characteristics of speech as process outcomes for both groups, and the numbers of photos and memory task scores as process outcomes for the intervention group. The results showed that verbal fluency improved significantly for the intervention group compared to the control group. Previous studies have reported that conversation-based interventions with trained interviewers impacted positively on verbal fluency performance (Cerino et al., 2019; Dodge et al., 2015).

Two of the common points regarding the conversational protocol of the previous studies as compared to that of the PICMOR program are that 1) topics are set, such as “childhood memories,” “hobbies,” “siblings and parents,” and “movies/books,” and 2) a daily picture prompt is used. The intention in using these topics and prompts was to stimulate the conversation to get spontaneous responses in order to take full advantage of this synthetic aspect of conversation. Differences between the two conversational protocols include the following: 1) the role of participants in the previous studies was that of a speaker (answering the questions asked by interviewers), while the PICMOR program participants were both speakers and interviewers (alternating between the two); 2) the amount of speech was regulated by the trained human interviewers in the previous studies but by the robot moderator in the PICMOR program; 3) pictures were provided by the researchers in the previous studies, while photos were taken by the participants in the PICMOR program; 4) only the PICMOR program had a recall phase.

Regarding trial design, conversation length was a common factor among all the studies, with 30 min for each. In terms of duration and frequency, web-based conversational interventions were more intensive, lasting for 6 weeks and made daily, while the PICMOR program lasted for 12 weeks and were weekly. Considering the common points and differences, verbal fluency improvement may have resulted from spontaneous speaking, where the amount of such speech was regulated by the protocol. Even though the PICMOR program was less intensive, the preparation of topics and taking photos beforehand and the asking questions and recalling the photos while conversing may have increased concentration and engagement so as to amplify the intervention effect. Further investigation into the essential parts of the protocol is needed.

### Verbal Fluency

Verbal fluency—more precisely, letter fluency, or the ability to produce words starting with a certain letter—improved for the intervention group compared to the control group. Tests of verbal fluency discriminate well between people with normal cognitive function and mild Alzheimer’s disease (Henry et al., 2004) and are, therefore, used for preliminary diagnosis of dementia in clinical settings. Verbal fluency draws on both executive functions and language abilities. Although verbal fluency declines with age, the slower processing masks the enhancement of letter fluency during the transition from youth to middle age. In one study, an older group performed better than a younger group on letter fluency after controlling for the decline in processing speed (Elgamal et al., 2011). This implies that verbal fluency is a trainable ability that should improve through the life course, and such improvement was indeed observed through the relatively short-term intervention in this study. The theory of cognitive reserve, where brain reserve is related to either the brain’s anatomical substrate or adaptability of cognition (Stern, 2013; Amieva et al., 2014), suggests that more brain reserve helps people tolerate more neuropathology without cognitive or functional decline, and therefore they develop dementia more slowly than do people without brain reserve (Stern, 2012). Improvement of verbal fluency in advance, which declines significantly at the onset of dementia, may gain some time to go below the level of dementia in later life.

### Linguistic Characteristics of Conversation

Linguistic ability is significantly associated with cognitive functions (Snowdon et al., 1996; Kemper et al., 2001). Our results show significant differences in evenness and the amount of information in conversations between the intervention and control groups even while the number of words spoken did not differ (Figure 4). This indicates that PICMOR can both induce participants to speak more and include much more information in their speech while keeping the amount of speech constant. Evenness in speech-sharing is important for conversation as intervention, as it entails balanced use of verbal comprehension and verbal production. In our study, some participants in free conversation (i.e., the control group) tended to speak much more and others less (Figure 3B), suggesting that temperamentally more talkative participants gain more skills at verbal production than verbal comprehension, and vice versa. In our intervention program, all participants had the opportunity to engage in verbal comprehension (listening) and verbal production (speaking), and the robot prompted participants who had had less speech and suppressed those with more speech, fostering evenness in conversation and possibly the higher verbal fluency scores. Moreover, the type–token ratio in the intervention group was higher than that in the control group. This is because participants in the intervention group took photos and made a presentation about things related to the photos, and then discussed them with each other. Furthermore, these participants would compose sentences effectively to pack more information into their speech. Previous studies used “idea density” as a similar index representing the amount of information in sentences and predicting cognitive function (Snowdon et al., 1996). Likewise, this intervention may increase verbal fluency.

### Classified Utterances of Group Conversation

Using the data of classified utterances, our analysis at the aggregate level found that the intervention group had higher percentages of utterances relevant to the Coimagination method (characterized as topic provision, questioning, and replying), even in the (Higher and Lower) subgroups, with a smaller total number of utterances in the intervention group. No clear correlations were found at the individual level. These results suggest that the intervention created an environment in which even those who speak less can talk relatively more about relevant things. The results also provide the following possible explanation of the mechanism of improvement of verbal fluency through our intervention program. First, by talking about their own topics (providing topic tags), participants retrieved vocabulary they had already had. Second, by actively listening to and asking questions about topics provided by other participants (question tag), the vocabulary triggered by other participants’ utterances was retrieved. Third, by actively listening to and answering questions about their own topics (reply tag), the vocabulary that they had had was further retrieved.

### Comparison of Intervention and Control Groups

The scores of both groups improved significantly after the Logical Memory I and II and Digit Symbol tests. The major reason may be that both intervention and control conditions included active group conversation. Another reason may be that the tests had learning effects. The trial will be conducted with a less active control condition, without group conversation, to clarify the main reason for these improvements.

The interesting point is that both groups engaged in conversations, but the manner of participation was different. This might have led to the difference in verbal fluency, which was reported to improve in previous conversational intervention studies. The implication here is that the manner of participation in conversations is a key to the gaining of cognitive benefits from them. This supports research gaps addressed, that the lack of conversation intervention quantification in the existing studies is a problem, and that controlling the amount of speech for intensity management is effective.

### Applications

PICMOR is applicable to practice and measurement support using methods with group sessions, such as cognitive stimulation therapy and group reminiscence therapy. Methods with group sessions generally require at least one trained facilitator per group, leading to increased training and hiring costs, that is, a scalability problem. PICMOR may increase scalability by obviating the need for human facilitator per group, at least for healthy older adults. A human instructor can remain to support participants who have special needs. With more robots, several group conversations can be coordinated by one human instructor operating multiple robots. In addition, building robots should also become cheaper at increased scale.

### Limitations and Future Work

This study was of relatively short length and held infrequent (i.e., weekly) sessions. Thorough investigation of the demonstrated effect of PICMOR warrants a longer study with more frequent sessions: for example, two or three times a week for two years, as in FINGER, the multi-modal lifestyle intervention study (Ngandu et al., 2015). This should also increase the visibility of the effects. A follow-up study is planned to investigate whether PICMOR may slow down cognitive decline and delay dementia for years. While the verbal function improvement is certainly an effect of conversation, some of the effects of PICMOR may be caused not by group conversation but by using photos and robots. The purpose of this study is to propose an effective, efficient, and reproducible intervention program in which group conversation characterized by balanced speech production is realized. The effect of each item should be further studied to make clear which aspect of PICMOR is essential for positive effects to occur.

## Data Availability

The datasets presented in this article are not readily available because a joint research agreement is required for data sharing, but are available from the corresponding author on reasonable request. Requests to access the datasets should be directed to MO-M, mihoko.otake@riken.jp.

## Supplementary Material

**Supplementary Table 1** The numbers of four types of utterances and their sum per participant.
